# Genome-wide identification and expression profiling of the auxin response factor (ARF) gene family in physic nut

**DOI:** 10.1371/journal.pone.0201024

**Published:** 2018-08-01

**Authors:** Yuehui Tang, Xinxin Bao, Kun Liu, Jian Wang, Ju Zhang, Youwei Feng, Yangyang Wang, Luoxiao Lin, Jingcheng Feng, Chengwei Li

**Affiliations:** 1 Key Laboratory of Plant Genetics and Molecular Breeding, Zhoukou Normal University, Henan, Zhoukou, China; 2 Henan Key Laboratory of Crop Molecular Breeding and Bioreactor, Henan, Zhoukou, China; 3 School of Journalism and Communication, Zhoukou Normal University, Henan, Zhoukou, China; Huazhong University of Science and Technology, CHINA

## Abstract

Auxin response factors (ARF) are important transcription factors which mediate the transcription of auxin responsive genes by binding directly to auxin response elements (AuxREs) found in the promoter regions of these genes. To date, no information has been available about the genome-wide organization of the ARF transcription factor family in physic nut. In this study, 17 *ARF* genes (*JcARFs*) are identified in the physic nut genome. A detailed investigation into the physic nut *ARF* gene family is performed, including analysis of the exon-intron structure, conserved domains, conserved motifs, phylogeny, chromosomal locations, potential small RNA targets and expression profiles under various conditions. Phylogenetic analysis suggests that the 17 JcARF proteins are clustered into 6 groups, and most JcARF proteins from the physic nut reveal closer relationships with those from *Arabidopsis* than those from rice. Of the 17 *JcARF* genes, eight are predicted to be the potential targets of small RNAs; most of the genes show differential patterns of expression among four tissues (root, stem cortex, leaf, and seed); and qRT-PCR indicates that the expression of all *JcARF* genes is inhibited or induced in response to exogenous auxin. Expression profile analysis based on RNA-seq data shows that in leaves, 11 of the *JcARF* genes respond to at least one abiotic stressor (drought and/or salinity) at, as a minimum, at least one time point. Our results provide valuable information for further studies on the roles of *JcARF* genes in regulating physic nut's growth, development and responses to abiotic stress.

## Introduction

The plant hormone auxin plays a vital role in the regulation of plant growth during many developmental stages including vascular elongation, fruit development, lateral root initiation, leaf expansion and senescence, tissue and organ patterning, and flowering, and in response to abiotic stresses [1]. In the past few years, several studies have explored the rapid effects of auxin on gene expression and regulation [1–2]. For example, the overexpression of an auxin-responsive GST gene, *OsGSTU4*, enhanced tolerance to salinity and oxidative stresses in transgenic *Arabidopsis* plants, and *OsGRX8*, a member of plant-specific CC-type group of GRX proteins, is differentially expressed during abiotic stress conditions and phytohormone auxin [1–2]. Expression of the majority of candidate genes is potentially regulated by auxins, and their primary functions in controlling plant growth and development processes have been determined in *Arabidopsis* and other plants [1–2]. Among the products of these genes, auxin response factors (ARF) are a well-known group of proteins that regulate the expression of auxin responsive genes by binding to the sequence TGTCTC in the auxin response elements (AuxREs) within their promoter regions [3]. Most ARF proteins are characterized by a highly conserved N-terminal DNA-binding domain (DBD) that recognizes an AuxRE present in the promoters of auxin-responsive genes, a repression domain or activation domain in their middle region and a carboxyl-terminal dimerization domain (CTD), which participates in protein–protein interactions through the hetero-dimerization of Aux/IAA and ARF family members and the hetero- and homo-dimerization of ARF proteins [3–4].

Since the first *Arabidopsis ARF* gene, *ARF1*, was cloned and its function investigated [5], a series of ARF proteins has been identified and characterized, by means of functional genomics and genome-wide analysis studies, from various plant species including *Arabidopsis* [6], rice [6], banana [7], apple [8], maize [9], sweet orange [10] and grape [11]. Based on the similarities of their full-length amino acid sequences and their topology, ARF family members are classified into six groups, namely I (AtARF3/4-like), II (AtARF10/16/17-like), III (AtARF1/2-like), IV (AtARF5-like), V (AtARF6/8-like), and VI (AtARF7/19-like) [9].

Genetic and molecular studies in *Arabidopsis* and other plant species have suggested that ARF proteins are key factors in regulatory networks controlling plant growth and development, based on the phenotypes of loss-of-function and gain-of-function mutants [12–18]. For example, the *arf3arf4* double mutant shows reduced abaxial identity in all lateral organs, including leaves [1]. *AtARF10* and *AtARF16* have been reported to be involved in lateral root formation, and the *arf10arf16* double mutant exhibits a strong auxin phenotype that results in the absence of lateral root formation, while this is not found in either the *arf10* or the *arf16* single mutant [7, 12]. An *Atarf7* mutation impairs the hypocotyl response to blue light and reduces the auxin response [13], and the *AtARF8* loss-of-function mutation affects hypocotyl elongation and auxin homeostasis [14]. The *AtARF7*/*AtARF19* double mutant shows aberrant lateral root formation and abnormal gravitropism in both hypocotyls and roots [15]. *AtARF10*, which is a target of *microRNA160*, functions in the regulation of seed germination and post-germination processes. In tomato, *SlARF3* is involved in the formation of trichomes and epidermal cells [16]. In addition, *ARF* genes participate in responses to abiotic stresses. For example, *OsARF16* has been found to be involved in the adaptive response to phosphate starvation response stress in rice [17]. *OsARF12* has been shown to play a role in iron homeostasis [18]. Although *ARF* genes thus have important roles in multiple aspects of plant growth and development, these gene families remain relatively poorly characterized in perennial species, especially members of the Euphorbiaceae. In addition, the regulatory mechanisms mediated by *ARF* gene products in perennial plants are not completely understood, and much remains to be learned about their roles in other plants. Identification of ARF proteins from perennial plants is therefore a necessary step in formulating more accurate hypotheses related to their functions in growth and development.

Physic nut (*Jatropha curcas* L.) is a multipurpose, perennial large shrub belonging to the Euphorbiaceae [19]. Its abilities to be resistant to drought and salt and to grow easily in barren soil, the high oil content of its seeds, and its adaptation to a wide range of agro-climatic conditions, suggest that physic nut has promise as a renewable source of biofuel [19–20]. Since physic nut genome data are now available [21], this species provides an opportunity for genome-wide annotation, classification and comparative genome research. To date, no detailed systematic investigation of the ARF family has been performed in physic nut. In the present study, a total of 17 putative *JcARF* genes in the physic nut genome were identified and characterized. Firstly, we performed a detailed bioinformatics analysis of the phylogeny, exon-intron structure, conserved domains, conserved motifs, chromosomal locations and potential small RNA targets of *JcARF* genes. Secondly, we analyzed the patterns of expression of these genes under normal growth conditions and in response to IAA, drought and salinity stresses by means of RNA-seq or quantitative real-time PCR (qRT-PCR). Taken together, these data will provide a foundation for future functional investigation of the ARF family in physic nut.

## Materials and methods

### Identification of ARF family members in physic nut

We used two strategies to search for members of the ARF family in the physic nut genome. Firstly, BlastP searches were performed against the physic nut genomics databases of the Kazusa DNA Research Institute (http://www.kazusa.or.jp/jatropha/) and the NCBI genome database (available from DDBJ/EMBL/GenBank under the accession number AFEW00000000), using ARF proteins from each group identified in *Arabidopsis* and rice as query sequences. Secondly, the ARF domain (PF06507) protein sequences were used as query sequences to carry out BlastP searches of the PFAM protein family database in order to identify the *JcARF* genes of physic nut. Furthermore, the domain analysis programs in SMART (http://smart.embl-heidelberg.de/) and PFAM (http://pfam.sanger.ac.ck/) were used to check whether each physic nut JcARF protein sequence was likely to be an ARF protein.

### Analysis of gene structure, conserved domains and motifs

The physic nut genome and the open reading frames of *JcARF* genes were downloaded from the Kazusa DNA Research Institute (http://www.kazusa.or.jp/jatropha/) and NCBI websites (https://www.ncbi.nlm.nih.gov/); the latter data are available from DDBJ/EMBL/GenBank under the accession number AFEW00000000). Gene structure was examined using the online Gene Structure Display Server GSDS 2.0 (http://gsds.cbi.pku.edu.cn/) by comparing open reading frame (ORF) sequences with the corresponding genomic sequences. MEME Suite Version 4.12.0 (http://meme-suite.org/tools/meme) was used to analysis the conserved motifs of JcARF proteins according to the following parameters: site distribution was set at zero or one occurrence per sequence (thus each sequence was allowed to contain at most one occurrence of each motif), the number of motifs to be found was 14 and motif width should be between 10 and 200. The DNAMAN6.0 software package was used to examine the conserved domains of JcARF proteins.

### Phylogenetic analyses, chromosomal locations and small RNAs target prediction

The protein sequences of the rice ARF family were downloaded from the Phytozome website (https://phytozome.jgi.doe.gov/pz/portal.html). Those of the *Arabidopsis* ARF family were obtained from the *Arabidopsis* information resource database (TAIR, http://www.arabidopsis.org/). ClustalX was used to carry out a multiple sequence alignment of ARF proteins [22]. A phylogenetic tree was constructed by the bootstrap method using MEGA6 with the following parameters: number of bootstrap replicates was 1000 and gaps/missing data treatment was complete deletion [3]. Chromosomal locations of members of the *JcARF* gene family were obtained from the physic nut genome database. Map distances in cM were calculated using the maximum likelihood mapping algorithm and the Kosambi mapping function [21]. The MapChart software package was used to draw the linkage maps for *JcARF* genes. The MiRanda software package (http://www.miranda-im.org/) was used to identify the small RNA target sites in *JcARF* genes according to the method described by Turner [23]. In addition, the sense orientation of each target gene sequence was compared with the reverse complement of the physic nut miR160, 167 and TAS3 sequences, and the potential target gene mRNAs that aligned closely with these miRNAs were identified.

### Plant materials and treatments

Physic nut seeds were collected from Guizhou province, China and planted on farmland in Guangzhou (113.3°E, 23.1°N), Guangdong province, China. Roots, stem cortex and leaf tissue of 20-day-old seedlings, and seeds at 14 days (the early developmental stage S1) and 35 days (the filling and maturation stage S2) after pollination, were used to analyze the patterns of expression of *JcARF* genes in physic nut. For IAA treatment, the fourth leaves of 25-day-old seedlings grown at 25°C under 16/8 h (light/dark) conditions in a growth chamber were incubated for 0 h, 1 h, 6 h and 12 h in either MS nutrient solution, as a control, or MS nutrient solution containing 15 μM indole-3-acetic acid (IAA). Leaves collected 0 h, 1 h, 6 h and 12 h after the start of IAA treatment were stored at -80°C for further analysis. Drought and salinity stress treatments were performed as described by Tang et al. (2016) [24]. The salinity and drought treatments were begun at the six-leaf stage (eight weeks after germination). For the salinity treatment, seedlings were irrigated daily with Hoagland solution plus 100 mM NaCl. For the drought treatment, irrigation was withheld. Leaves were sampled after 2 days, 4 days and 7 days of drought stress and after 2 hours, 2 days and 4 days of salinity stress. Samples were frozen immediately in liquid nitrogen and stored at -80°C prior to digital gene expression and quantitative PCR (qRT-PCR) analysis The raw data were submitted to the sequence read archive (SRA) at NCBI (accession number PRJNA244896 for salinity stress; accession number PRJNA257901 for drought stress).

### RNA isolation, cDNA synthesis and qRT-PCR

A HiPure plant RNA mini kit (Magen, http://www.magentec.com.cn/) was used to isolate total RNA from different physic nut tissues. A DNase on-column kit (Magen, http://www.magentec.com.cn/) was used to remove contaminating DNA from RNA extracts. First strand cDNA synthesis was performed with a PrimeScript™ II 1st strand cDNA synthesis kit (TAKARA, Japan). qRT-PCR was carried out using a LightCycler^®^ 480 Real Time PCR system (Roche, CA, UAS) and SYBR^®^ *Premix* EX Taq^TM^ II (TAKARA, Japan). All kits were used according to the manufacturers’ instructions. All gene-specific primers were designed using the Primer Premier 5.0 software package (http://www.premierbiosoft.com/primerdesign/) according to the following criteria: PCR amplicon lengths of 100–200 bp, Tm of 60± 1°C and GC contents of 45–60%, based on the sequences of the C-terminal regions of *JcARF* genes. The primers employed are listed in S1 Table. The 2^-ΔΔCT^ method was used to calculate relative expression levels, and *JcActin* from physic nut was used as the reference gene. All experiments included three biological replicates, and two technical replicates of each.

### Statistical analysis

Statistical analysis of the data was done, using the SAS software package, by means of the Duncan multiple range test [25].

## Results

### Identification of *JcARF* genes in physic nut

To identify the *JcARF* genes of physic nut, we performed BlastP searches against the physic nut genomics databases using the ARF domain and all ARF proteins previously identified in rice and *Arabidopsis*. In total, genes encoding 17 putative ARF transcription factors were identified in physic nut, and these genes were provisionally named *JcARF1* to *JcARF17* based on their positions (from top to bottom) on physic nut linkage groups (LGs) 1 to 11. The open reading frame (ORF) lengths of *JcARF* genes ranged from 1074 bp (*JcARF7*) to 3402 bp (*JcARF17*), thus potentially encoding proteins of 357–1133 amino acids, and their accession numbers in GenBank are listed in Table 1. The predicted molecular masses of the 17 deduced JcARF proteins ranged from 40.6 to 126.4 KDa, and the theoretical pIs were between 5.09 and 7.55 (Table 1).

**Table 1:** Summary of *JcARF* genes encoding ARF proteins in physic nut.

Genes	Gene ID	protein length (aa)	pI	MW	Location	Domain
				(kDa)		
*JcARF1*	JCGZ_05508	707	7.55	77.8	LG2	DBD, ARF, CTD
*JcARF2*	JCGZ_05557	780	6.32	86.1	LG2	DBD, ARF, CTD
*JcARF3*	JCGZ_04659	899	6.15	99.5	LG3	DBD, ARF, CTD
*JcARF4*	JCGZ_21491	734	7.11	80.8	LG3	DBD, ARF
*JcARF5*	JCGZ_19776	1115	6.11	123.1	LG4	DBD, ARF, CTD
*JcARF6*	JCGZ_12025	591	5.74	65.1	LG4	DBD, ARF
*JcARF7*	JCGZ_26375	357	6.65	40.6	LG5	DBD, ARF
*JcARF8*	JCGZ_26377	854	6.21	95.3	LG5	DBD, ARF, CTD
*JcARF9*	JCGZ_16506	703	6.56	77.2	LG6	DBD, ARF, CTD
*JcARF10*	JCGZ_13849	713	5.78	79.4	LG8	DBD, ARF, CTD
*JcARF11*	JCGZ_14244	705	6.00	78.4	LG8	DBD, ARF, CTD
*JcARF12*	JCGZ_09784	830	5.90	93.2	LG9	DBD, ARF, CTD
*JcARF13*	JCGZ_20425	695	6.24	77.7	LG9	DBD, ARF, CTD
*JcARF14*	JCGZ_00307	680	5.76	75.9	LG10	DBD, ARF, CTD
*JcARF15*	JCGZ_20054	686	6.01	75.5	LG10	DBD, ARF, CTD
*JcARF16*	JCGZ_08238	945	5.09	104.1	LG11	DBD, ARF, CTD
*JcARF17*	JCGZ_07520	1133	6.04	126.4	LG11	DBD, ARF, CTD

### Phylogenetic relationships and structures of the *JcARF* genes

**Fig 1:**
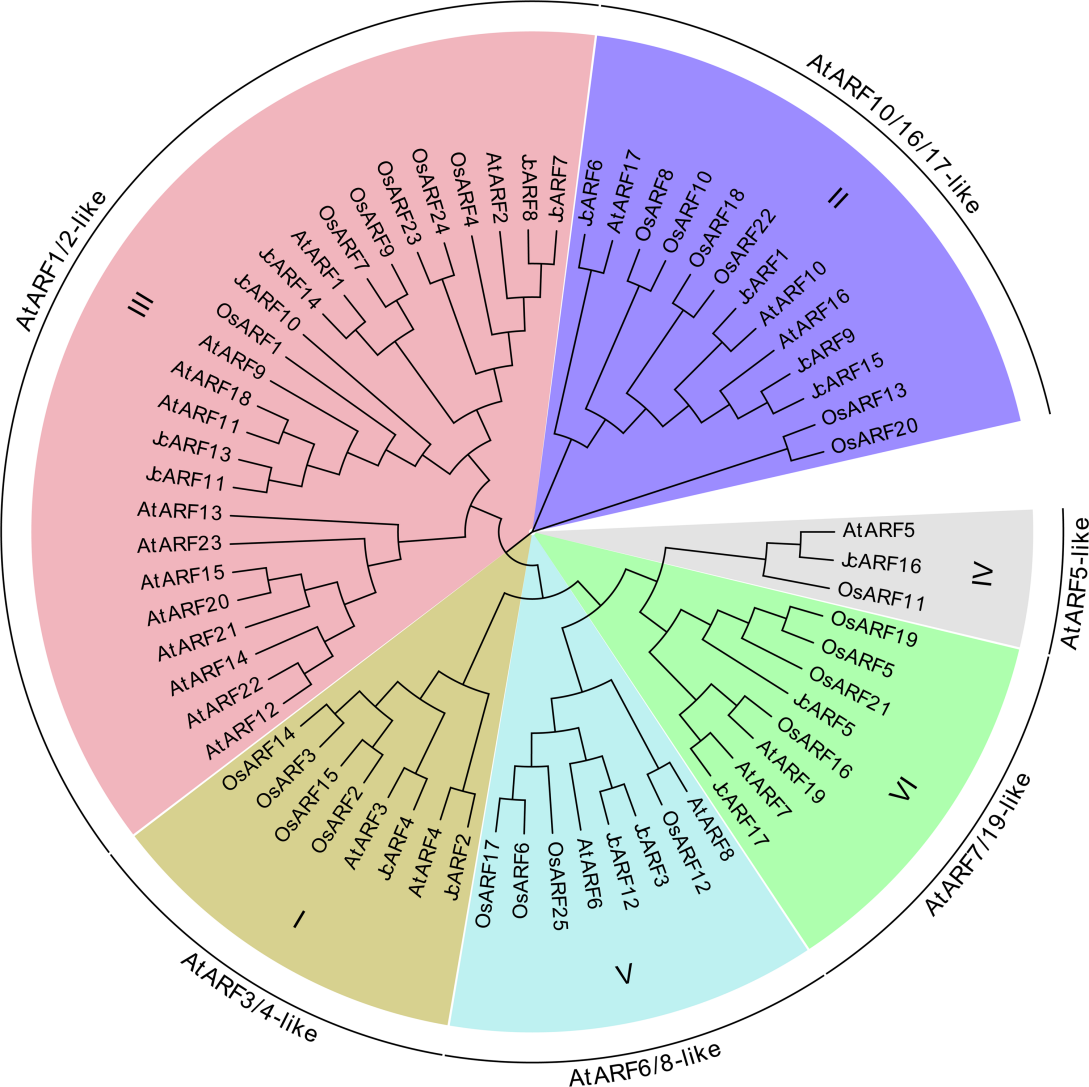
Phylogenetic relationships of *Arabidopsis*, rice and physic nut ARF proteins. The unrooted tree was constructed, using the MEGA6.0 program, by the neighbor-joining method. Bootstrap values were calculated for 1000 replicates.

To survey the evolutionary relationships between JcARF proteins from physic nut and previously-reported ARF proteins from the dicot *Arabidopsis* and the monocot rice [6], we constructed an unrooted phylogenetic tree using MEGA6.0 software with the Neighbor-Joining (NJ) method based on the full-length amino acid sequence similarity and topology of 17 JcARFs, 23 AtARFs and 25 OsARFs. The NJ tree indicated that the ARF family proteins of rice, *Arabidopsis* and physic nut could be divided into 6 groups, designated I (AtARF3/4-like), II (AtARF10/16/17-like), III (AtARF1/2-like), IV (AtARF5-like), V (AtARF6/8-like) and VI (AtARF7/19-like) (Fig 1). Eight members were assigned to group I (2 members from physic nut), 13 members to group II (4 from physic nut), 25 to group III (6 from physic nut), 3 to group IV (1 from physic nut), 8 to group V (2 from physic nut) and 8 were assigned to group VI (2 from physic nut). The results also indicated that most of the JcARF proteins were closer to the members of the AtARF family than to members of the OsARF family, as shown in the phylogenetic tree (Fig 1). For example, JcARF2 and JcARF4 were clustered with AtARF3 and AtARF4 in group I (AtARF3/4-like), while OsARF2, OsARF3, OsARF14 and OsARF15 branched off into a separate clade in group I.

**Fig 2:**
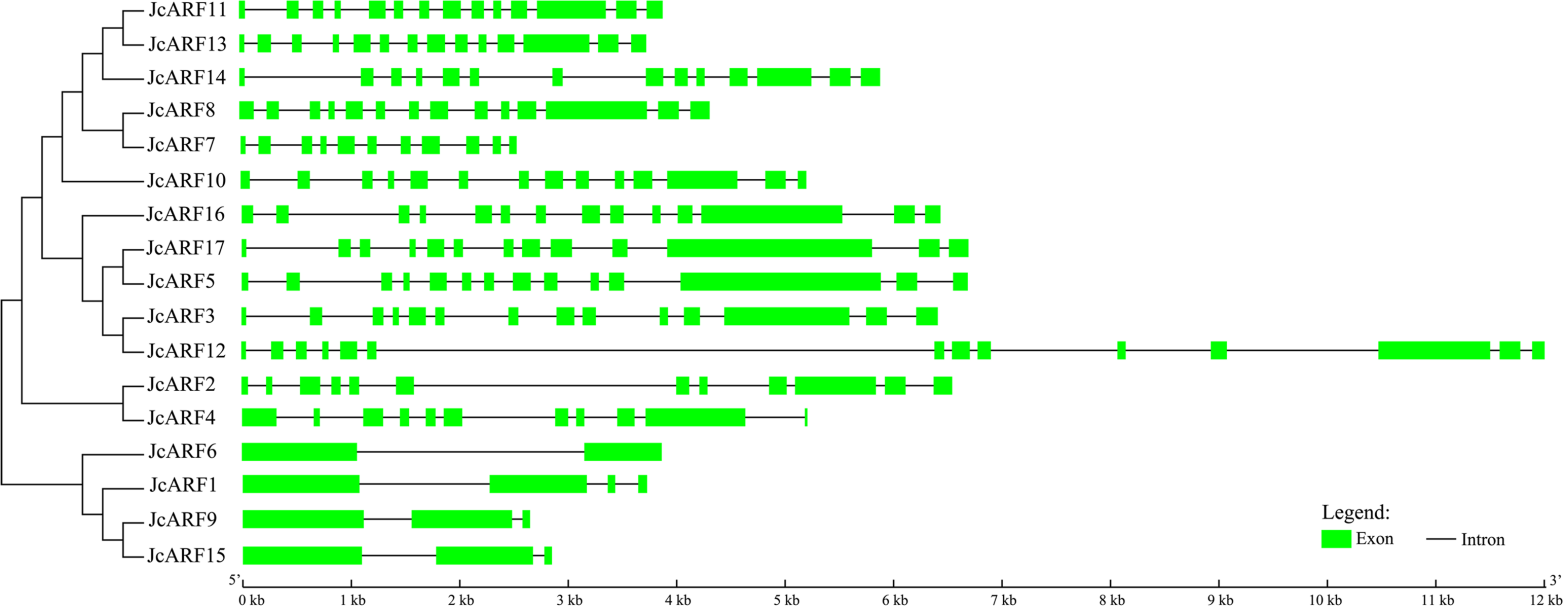
Exon-intron structures of *JcARF* genes. Exons and introns are shown as green boxes and thin lines respectively.

Exon/intron analysis can provide valuable information concerning evolutionary relationships among plant taxa. We therefore analyzed the exon/intron structures of *JcARF* genes. The results showed that all *JcARF* genes had one or more introns (Fig 2), and revealed the very highly conserved exon/intron splicing arrangement that has previously been reported for *Arabidopsis*, rice, banana, poplar, grape and maize [4, 6, 7, 9, 26]. In general, most of the *JcARF* members within a given group possessed a similar exon-intron structure in terms of intron numbers and exon length. For example, the *JcARF* genes in group II had one or three introns, whereas all group V members had thirteen introns in their ORF regions. This conserved exon/intron structure shared within each group provided further support for the evolutionary relationship and classification of the physic nut ARF family members identified in this study.

### Conserved domains and motifs in JcARF proteins

**Fig 3:**
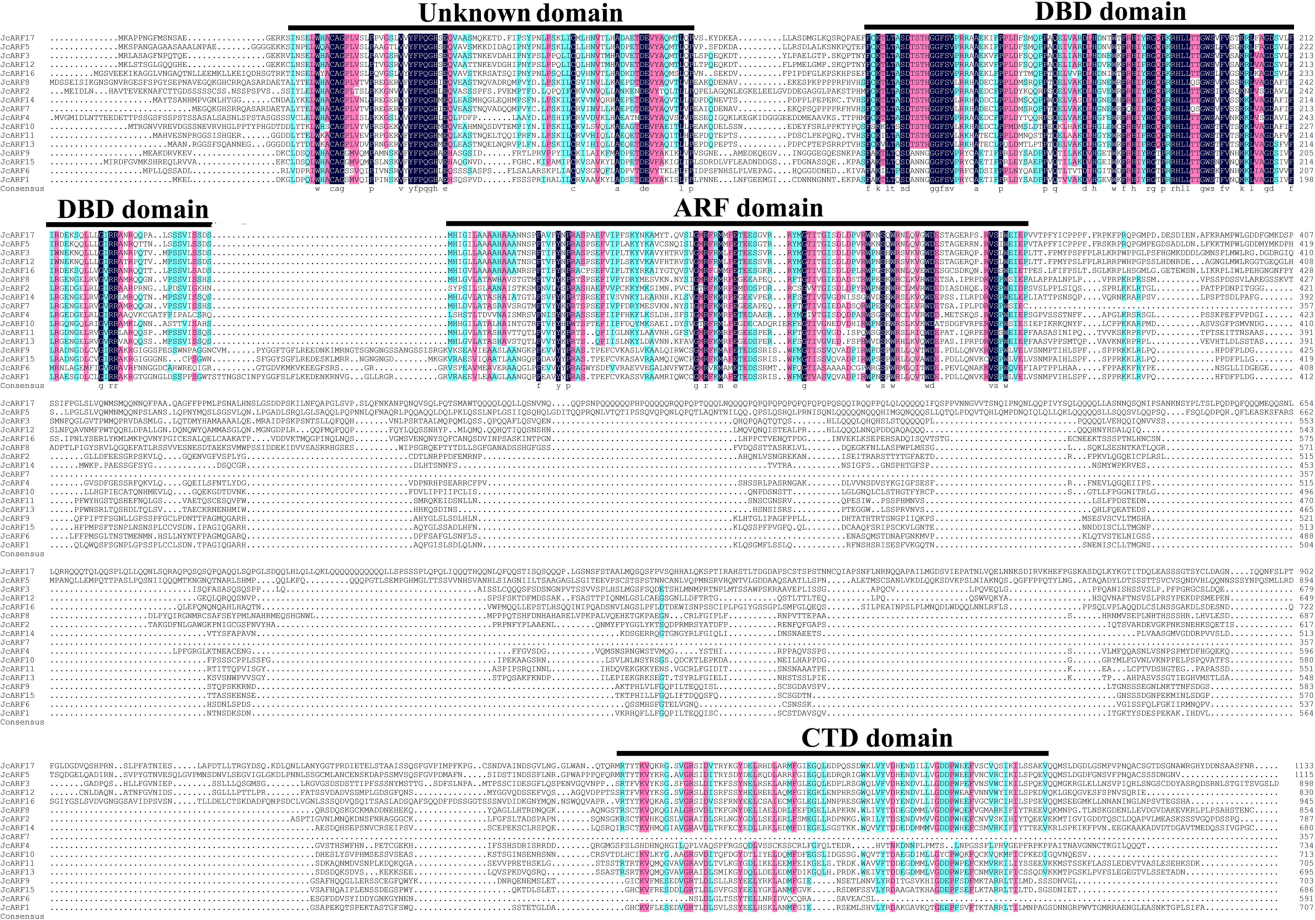
Multiple sequence alignment analysis of physic nut ARF proteins carried out using the DNAMAN6.0 program. Conserved domains in JcARF proteins are underlined and indicated.

To examine the structural domain features of the products of the 17 physic nut *JcARF* genes, a multiple sequence alignment analysis of JcARF proteins was performed using the DNAMAN6.0 program. The results indicated that all of the JcARF proteins contained a well-conserved DBD sequence of about 100 amino acids at the N-terminal region (Fig 3). Most JcARF proteins contained a CTD; the exceptions were JcARF4, JcARF6 and JcARF7 (Fig 3).

The middle region of the ARFs, which is nonconserved, has been shown to function as a transcriptional repression or activation domain [27–28]. Protoplast transfection assays showed that AtARF5, 6, 7, 8 and 19, which contain glutamine-rich (Q-rich) middle regions, function as activators. These assays also indicated that AtARF1, 2, 3, 4 and 9 are repressors; AtARF1 contains a middle region that is Proline/Serine/Threonine-rich (P/S/T-rich) [27–28]. Our sequence analysis of all 17 JcARF proteins revealed that the central regions of JcARF3, 5, 12, 16 and 17 were rich in Q, suggesting that these genes are probable transcriptional activators with roles in physic nut development; the functions of these genes need further investigation. In contrast, P/S/T-rich middle regions were found in JcARF2, 4, 8, 10, 11, 13 and 14, indicating that the products of these genes are possible transcriptional repressors (Fig 3).

**Fig 4:**
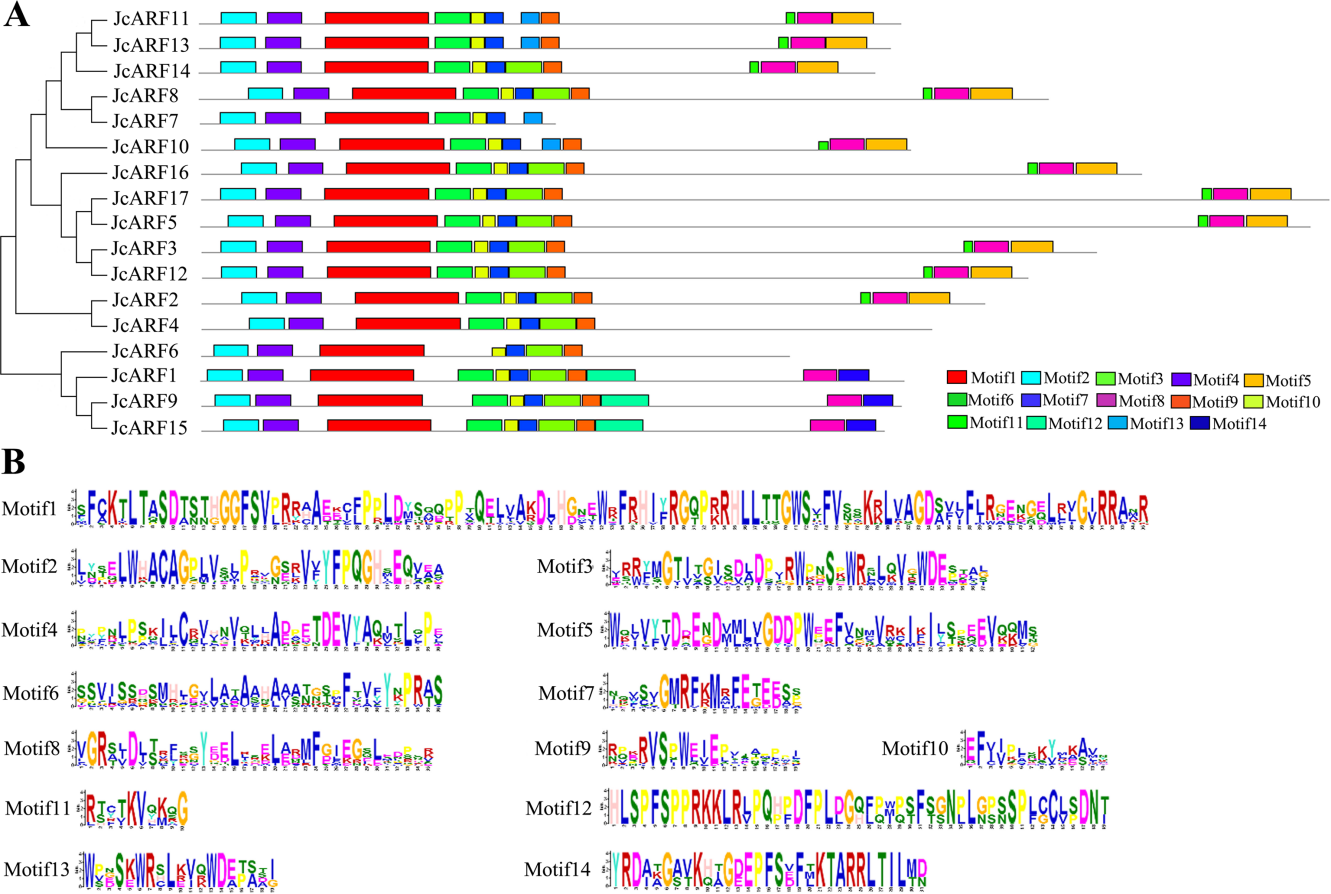
Conserved motifs in JcARF proteins. (A) Motifs were determined using MEME suite version 4.12. Grey lines represent the non-conserved sequence, and each motif is indicated by a colored box numbered at the bottom. (B) Motif logo, the amino acid composition of each motif.

We then analyzed the sequence features of JcARF proteins using MEME suite version 4.12. The results indicated the presence of 14 individual motifs in the 17 JcARF proteins; we named these motifs 1–14 (Fig 4). As predicted, the amino acid sequences of these motifs were highly conserved, like those of the homologous sequences detected in ARF proteins from rice, *Arabidopsis* and maize [6, 9], but the functions of most of these putative motifs were not clear because they lacked homologs in protein motif databases such as SMART and Pfam. The DBD sequence corresponded to motif 1, and the ARF domain was made up of motifs 3, 6, 7, 9 and 10. Motifs 5, 8, 11 and 14 corresponded to the CTD region. All predicted JcARF proteins contained motifs 1, 2, 4, 7 and 10 (Fig 4). Furthermore, most of the 14 conserved motifs found in JcARF proteins were clade-specifically distributed in the phylogenetic tree, implying functional similarities within the same group.

### Chromosomal localization of *JcARF* genes

**Fig 5:**
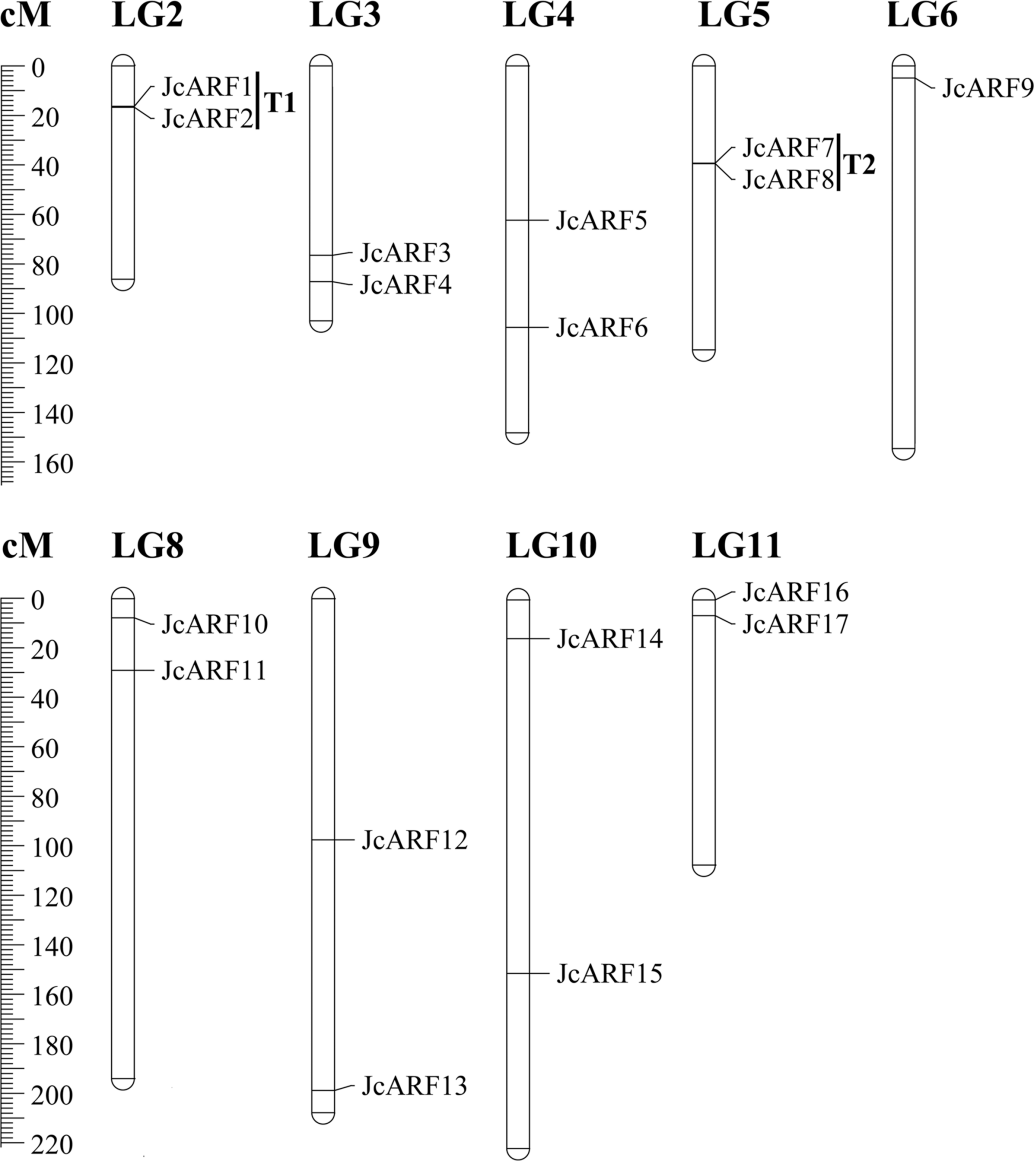
Distribution of *JcARF* genes on physic nut chromosomes according to the linkage map. In total, 17 *JcARF* genes were mapped to 9 linkage groups (LGs). The scale is in centiMorgans (cM). T, tandem duplication.

All 17 members from *JcARF* gene family were mapped onto the physic nut linkage groups (LGs) [21]; none of the genes mapped to LGs 1 and 7 (Fig 5). Of the remaining nine linkage groups, there were two *JcARF* genes on each of linkage groups 2, 3, 4, 5, 8, 9, 10 and 11, and one on LG 6. Most *JcARF* genes were on the upper and middle regions of the LGs. Tandem duplications, defined as tandem repeats that are located within 50 kb from each other or are separated by < 4 non-homologous spacer genes [29], were found among the members of the physic nut ARF family. The gene pairs present as tandem repeats (T) were T1 (*JcARF1* and *2*) and T2 (*JcARF7* and *8*), on LG 2 and LG 5 respectively (Fig 5).

### Prediction of potential targets for small RNAs in *JcARF* genes

TAS3 (trans-acting short interfering RNA, tasiRNA) and microRNAs (miR160, miR167) have been reported to target *Arabidopsis ARF* genes [30–32]. As these small RNAs and their targets are evolutionarily conserved across many plant species [33], we looked for potential small RNA targets in physic nut *JcARF* genes by using the miRanda software package. The results (S2 Table) indicated that 4 members of group II (*JcARF1*, *JcARF6*, *JcARF9* and *JcARF15*) were miR160 targets, 2 members of group V (*JcARF3* and *JcARF12*) were miR167 targets, and 2 members of group I (*JcARF2* and *JcARF4*) were TAS3 targets, whereas no target sites for small RNAs were detected in group III, IV, or VI. These results furnish further support for the identification and classification of the *JcARF* gene family described above.

### Expression profiles of *JcARF* genes in physic nut under normal growth conditions and in response to IAA treatment

**Fig 6:**
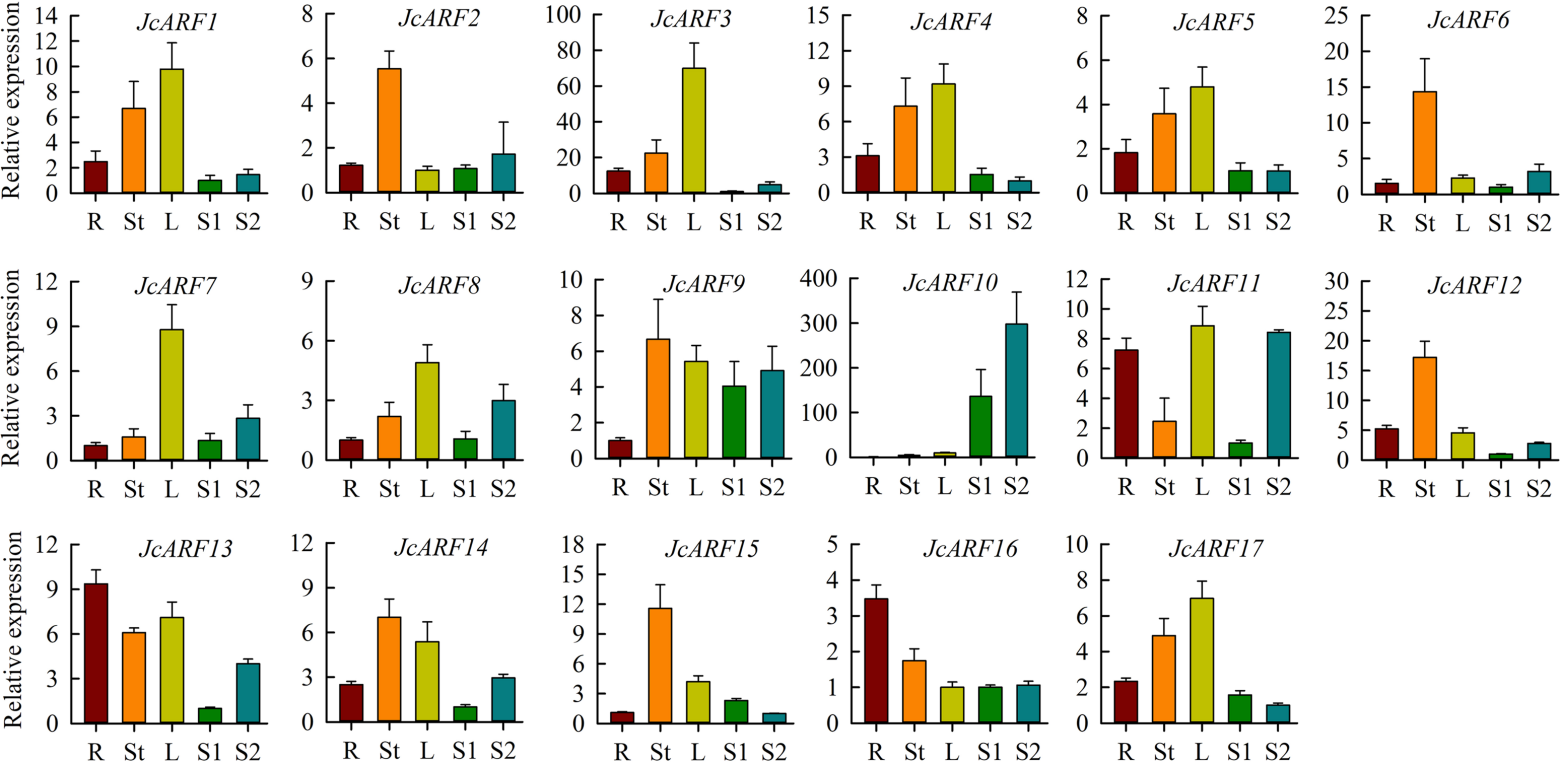
Expression profiles of *JcARF* genes in different tissues. qRT-PCR analysis of *JcARF* genes in roots (R), stem cortex (St), leaves (L), and seeds at 14 (S1) and 35 (S2) days after pollination. Relative mRNA expression was normalized to that of the reference gene *JcActin* (internal control). Bars show means ± SD of three biological replicates.

In order to clarify the roles of *JcARF* genes in physic nut growth and development, we examined the expression profiles of these genes in the roots, stem cortex, leaves, and S1 and S2 seeds under normal growth conditions by qRT-PCR. The results indicated that all *JcARF* genes were expressed in the different tissues tested in this study. Of the 17 *JcARF* genes, one (*JcARF16*), four (*JcARF2*, *6*, *12* and *15*), four (*JcARF3*, *7*, *8* and *17*), and one (*JcARF10*) showed their highest expression levels in root, stem cortex, leaves, and seeds respectively (Fig 6). Conversely, 7 *JcARFs* (*JcARF1*, *4*, *5*, *9*, *11*, *13* and *14*) had a relatively constitutive pattern of expression in all tissues tested (Fig 6), suggesting that these genes may play important roles throughout physic nut growth and development. In addition, five *JcARFs* (*JcARF8*, *10*, *11*, *13* and *14*) exhibited higher expression in seeds at the filling and maturation stage (S2) than in seeds at the early development stage (S1) (Fig 6).

**Fig 7:**
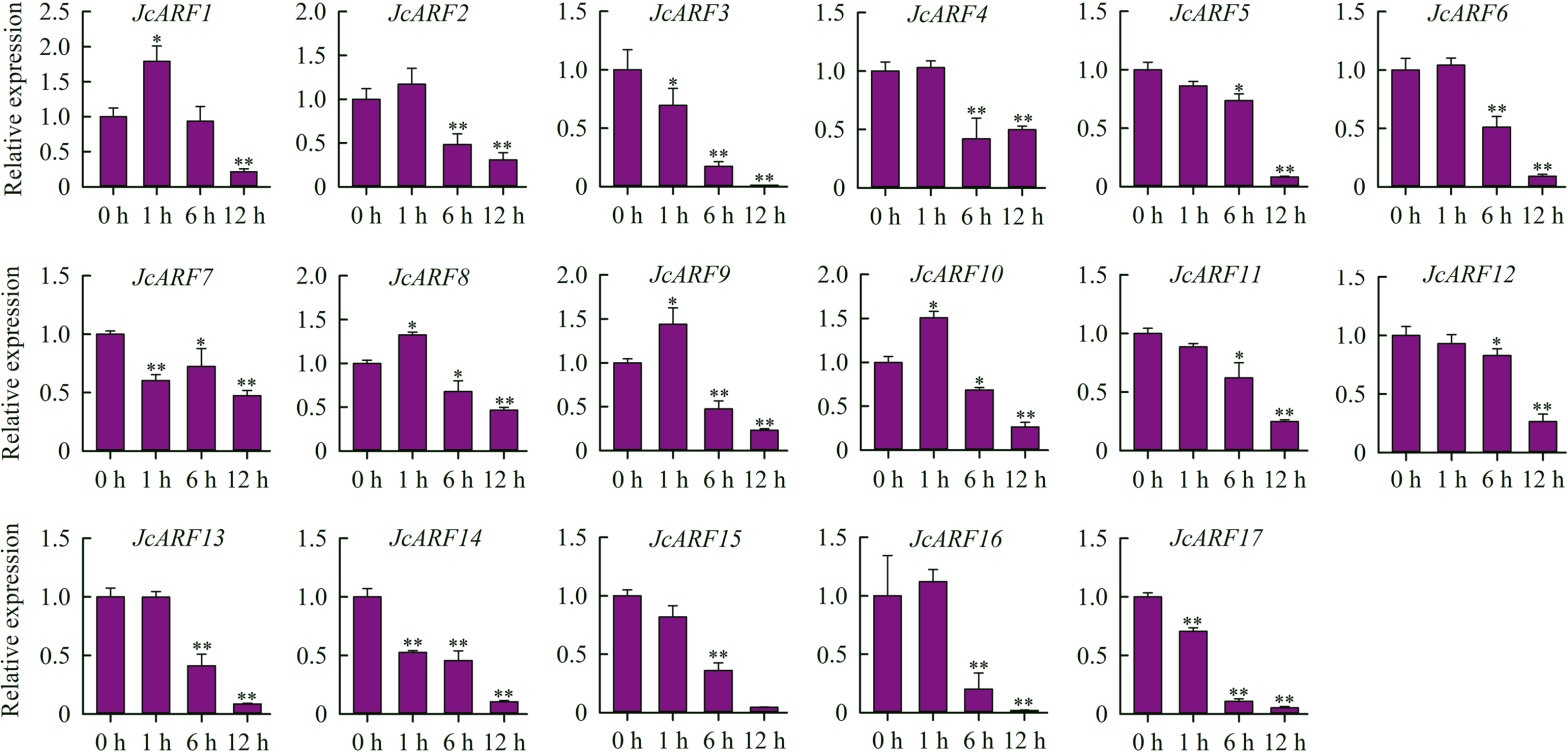
Expression analysis of *JcARF* genes in leaves in response to IAA, carried out by qRT-PCR. The fourth leaves of 25-day-old seedlings grown at 25°C under 16/8 h (light/dark) conditions in a growth chamber were incubated for 0 h, 1 h, 6 h or 12 h in either MS nutrient solution (control) or MS nutrient solution containing 15 μM indole-3-acetic acid (IAA). The *JcActin* gene was used as an internal control. The experiment included three biological replicates, each with two technical replicates. Values represent means of n = 6 ± SD (Duncan test: **, P < 0.01; *, P < 0.05).

It has reported that some ARF family transcription factors are involved in response to auxin [34]. We therefore explored the patterns of expression of *JcARF* genes in leaves 0 h, 1 h, 6 h and 12 h after the start of IAA treatment, using qRT-PCR. The results showed that five *JcARF* genes (*JcARF3*, *7*, *14*, *15* and *17*) were progressively down-regulated over time after IAA treatment (Fig 7). The expression of 16 *JcARF* genes (*JcARF2*, *3*, *4*, *5*, *6*, 7, 8, *9*, *10*, *11*, *12*, *13*, *14*, *15*, *16* and *17*) was suppressed in leaves after 6 h and 12 h of IAA treatment (Fig 7). The expression level of 8 *JcARF* genes (*JcARF4*, *5*, *6*, *11*, *12*, *13*, *15* and *16*) showed no obvious difference after 1 h of IAA treatment compared to the control, while 4 *JcARF* genes (*JcARF1*, *8*, *9* and *10*) were up-regulated by 1 h after the start of IAA treatment (Fig 7).

### Expression profiles of *JcARF* genes in physic nut under conditions of drought and salinity stress

**Fig 8:**
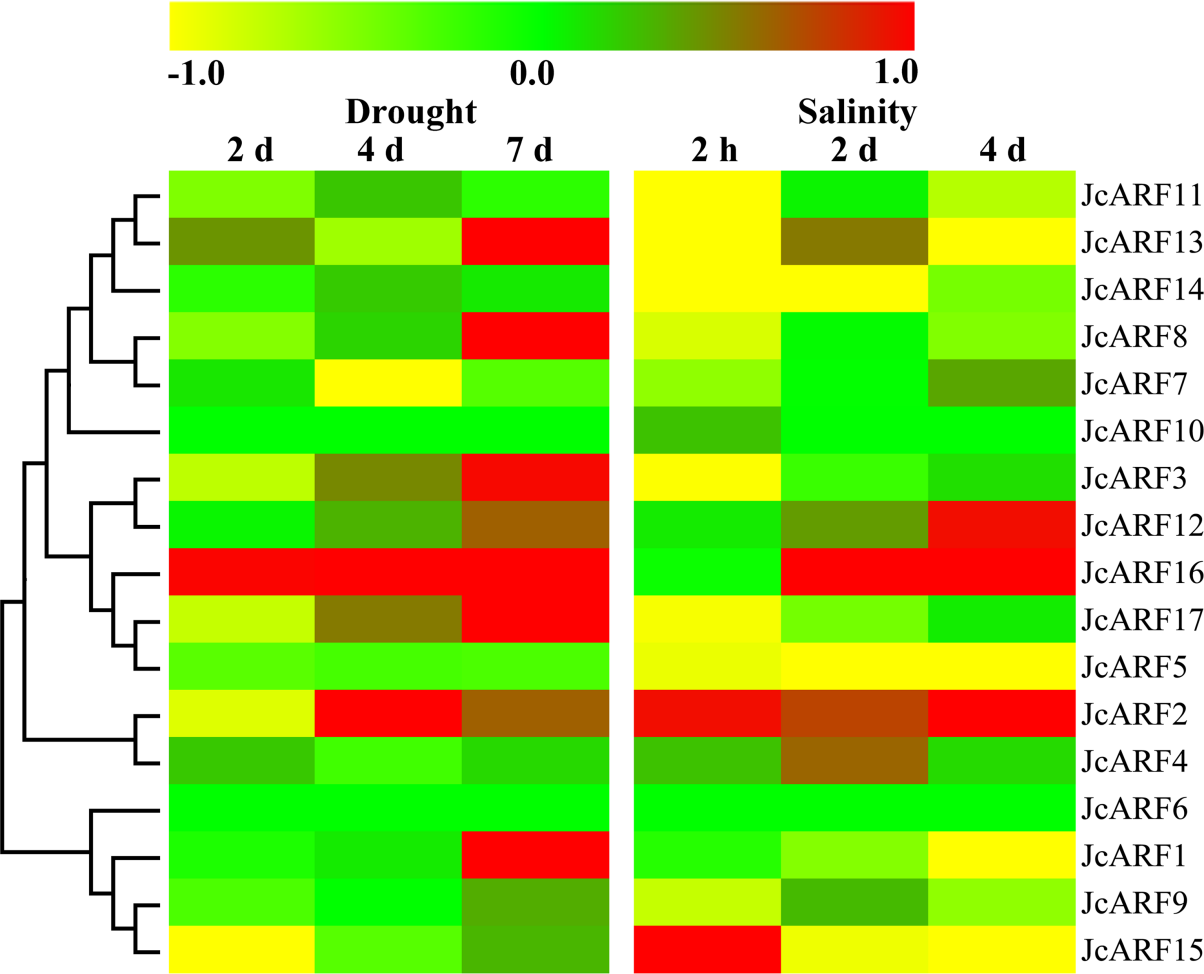
Expression analysis of 17 *JcARF* genes in leaves under drought and salinity stresses. Log_2_ based values (signal values for treatment/signal value for control) were used to create the heat map based on transcriptomic data for ARFs. The color scale (representing signal values) is shown at the top.

In recent years, increasing numbers of studies have shown that auxin acts as an integral part of plant responses to unfavorable abiotic stress [35–37]. Thus we examined the levels of expression of *JcARF* genes in leaves 2 d, 4 d and 7 d after exposure to drought stress [38] and 2 h, 2 d and 4 d after exposure to salinity stress [39], using a transcriptome sequencing technology (RNA-seq) approach (Fig 8 and S3 Table). The results indicated that 11 *JcARF* genes underwent at least a 2-fold increase or decrease in expression in response to at least one stress. Out of these 11 differentially expressed genes, four (*JcARF2*, *JcARF13*, *JcARF15* and *JcARF16*) showed the differential response at a minimum of one time point in response to both drought and salinity, four (*JcARF1*, *JcARF7*, *JcARF8* and *JcARF17*) responded only to drought stress, and three (*JcARF5*, *JcARF11* and *JcARF14*) responded only to salinity stress (Fig 8 and S3 Table).

**Fig 9:**
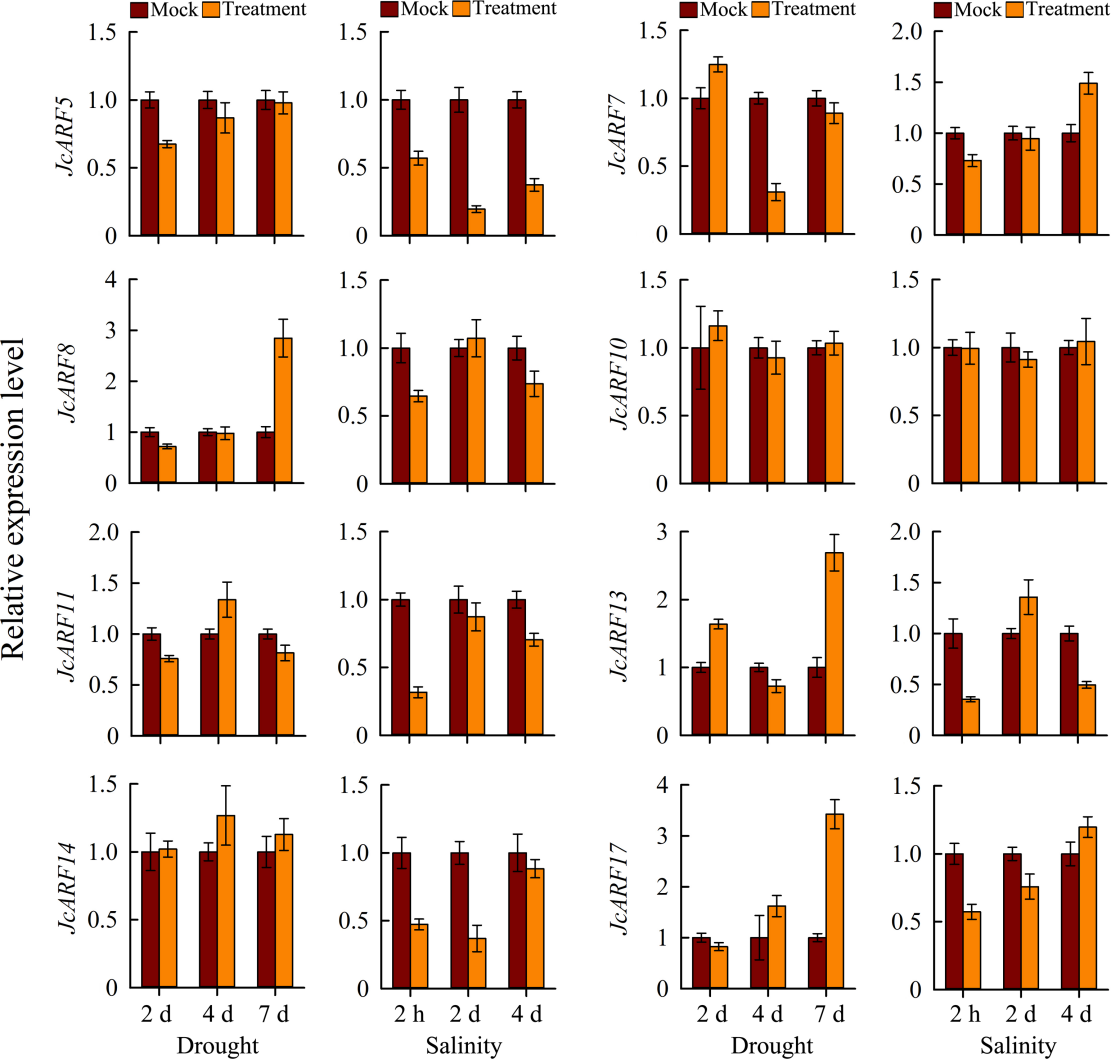
Expression profiles of selected *JcARF* genes. qRT-PCR analysis of selected genes in leaves after the onset of drought and salinity stresses. Relative expression was normalized to the reference gene *JcActin* as an internal control. Bars show standard deviations of the repeats. The experiment included three biological replicates, each with two technical replicates.

To verify the expression patterns of *JcARF* genes after drought and salinity stresses that we obtained from RNA-seq data, we analyzed the abundance of the transcripts of groups III (*JcARF7*, *JcARF8*, *JcARF10*, *JcARF11*, *JcARF13* and *JcARF14*) and VI (*JcARF5* and *JcARF17*) genes in leaves 2 d, 4 d and 7 d after exposure to drought stress, and 2 h, 2 d and 4 d after exposure to salinity stress, using qRT-PCR (Fig 9). The results were largely consistent with the abundances of their transcripts and the gene expression changes observed in the RNA-seq experiments, showing that the RNA-seq data were generally accurate.

## Discussion

Physic nut has been shown to be a salinity and drought-tolerant plant, a factor facilitating its widespread adoption for a variety of purposes [19]. However, the mechanisms by which physic nut respond to abiotic stresses have been poorly understand up to now. Members of the ARF family have been reported to be involved in the regulation of plant growth, development and response to abiotic stresses [13–18]. However, the identity and functions of physic nut *ARF* genes were previously unknown. Thus in order to shed light on the roles of *JcARF* genes in regulating plant growth, auxin signaling and transduction and response to abiotic stresses in physic nut, we delineated the major structural characteristics and expression profiles of ARF family genes in this species.

In this study, a total of 17 physic nut *JcARF* genes were identified. There were fewer members of the *JcARF* family in the physic nut genome (genome size 320 Mb) than in *Arabidopsis* (genome size 125 Mb), which has 23 ARFs, or rice (genome size 466 Mb), with its 25 ARFs [6, 21]. One possible explanation for the smaller number of *JcARF* genes may be that ARF family genes in the physic nut genome have not undergone a chromosomal segment duplication event during the early evolution of the species [21], whereas such duplications made major contributions to the expansion of both *Arabidopsis ARFs* and rice *ARFs* [6]. Similar to the situation in rice and *Arabidopsis*, physic nut ARF proteins could be divided into six well-delimited groups (Fig 1). The same number of ARF groups is also found in other plants [9]. Phylogenetic trees suggested that there were four JcARF proteins (23.5%, 4 of 17) in group II, whereas only three *Arabidopsis* AtARFs (13%, 3 of 23) were assigned to this group (Fig 1). This suggests that the genes of the group may have been either acquired in the physic nut or lost from the *Arabidopsis* lineage after divergence from the last common ancestor shared by *Arabidopsis* and physic nut.

The extent to which intron-exon patterns differ among plant species closely reflects the evolutionary relationships of these species. The exon-intron splicing arrangement and intron numbers in the *JcARF* genes in the physic nut genome were similar to those reported in *Arabidopsis* [6], rice [6] and maize [9]. For example, the members of physic nut group II had the smallest numbers of introns (1 to 3) (Fig 2). Similarly, group II genes from *Arabidopsis*, rice and maize have 1 to 3 introns [6, 9]. Motif analysis indicated that the conserved N-terminal DBD domain (Motif 1) was present in all JcARF proteins, indicating that JcARF transcription factors have been highly conserved in various plants over the course of evolution. Our results also suggested that each group had its own common motifs (Fig 4). These features in the conserved regions of ARF proteins have also been also found in other plants, such as *Arabidopsis*, maize and rice [6, 9]. Generally, most of the *JcARF* genes in a given group had similar gene structure and conserved motifs, which further supports their classification as described here and the evolutionary relationships among the groups.

Previous research has indicated that gene duplication events are important in the evolution and rapid expansion of gene families [40]. In our study, chromosomal localization analysis suggested that a tandem gene duplication has occurred in group III in physic nut, giving rise to *JcARF7*/*JcARF8* (Fig 5). Such duplications are also observed in rice, banana, *Arabidopsis* and maize [6–9]. Thus, we suspect that tandem gene duplication may have played a significant role in ARF family expansion in physic nut and other plants. These results are consistent with the finding that group III had the largest number of ARF proteins among the six groups.

The expression profile of a gene often has some connection with its function. To date, although the patterns of expression of *ARF* genes have been determined in other plants, such as rice, maize and *Arabidopsis* [6–9], there have been no detailed studies of the expression of physic nut *ARF* genes. In the present study, the expression profiles of *JcARF* genes in different tissues were examined using qRT-PCR. The analysis indicated that *JcARF7* expression was highest in leaves (Fig 6), and its *Arabidopsis* homolog *AtARF2* has been reported to regulate leaf senescence [41]. Thus we can deduce from the high levels of *JcARF7* transcription in leaves that it is likely to be involved in leaf senescence in physic nut. *AtARF5* is essential for embryonic root formation [1], and its homolog *JcARF16* was most highly expressed in root (Fig 6). The result suggests that *JcARF16* may play roles in root formation in physic nut. *AtARF7* has been shown to function in the regulation of leaf expansion [1], and its physic nut homolog *JcARF17* was preferentially expressed in leaves, suggesting a role in leaf development. *OsARF3* is known to function in regulating the response to auxin during de novo shoot regeneration [42], and its homolog *JcARF2* was most strongly expressed in stem cortex of all the organs tested; its expression was repressed in response to IAA treatment, indicating that it could play a role in mediating the auxin response during de novo shoot regeneration. Previous studies have suggested that *Arabidopsis AtARF8*, which is a target of miR167, plays important roles in regulating leaf growth and fruit development [1, 14, 32]. In our study, we identified an miR167 target site in the mRNA of its homolog *JcARF3* (S2 Table), and *JcARF3* expression was highest in leaves (Fig 6). These results indicated that *JcARF3* may be involved in leaf growth and be regulated by miR167, which may mediate a reduction in the abundance of *JcARF3* gene transcripts. The *AtARF3/4* genes have been reported to control leaf development and leaf polarity specification [43–44]. Their physic nut homolog *JcARF4* was most strongly expressed in leaves (Fig 6), suggesting that *JcARF4* may carry out similar functions to these *Arabidopsis* genes in growth and developmental processes. Overall, we speculate that *JcARF* genes may be involved in diverse aspects of developmental processes during the growth of physic nut, and their functions merit further study.

Since *ARFs* regulate the expression of auxin response genes, we determined the responses of *JcARF* genes to exogenous IAA treatment. It has been reported that expression of *CiARF3*, *8*, *12*, *15*, *17*, *19* and *OsARF5*, *14* and *21* significantly decreased under auxin treatment, whereas that of *AtARF4*, *5*, 19 and *OsARF1* slightly increased [6, 10]. In our study, expression of *JcARF* genes changed rapidly under exogenous IAA treatment compared with the control physic nut plants. For example, 16 of the 17 *JcARF* genes were significantly down-regulated in leaves after 6 h and 12 h of IAA treatment, and 4 *JcARF* genes were up-regulated after 1 h of IAA treatment (Fig 7). These results suggest a role for auxin in mediating *JcARF* gene expression in physic nut.

Studies focusing on genome-wide expression analysis in various plants have revealed that the abundance of some *ARF* genes transcripts is altered under abiotic stress conditions [45–46]. For example, in banana, most *ARF* genes have been reported to alter expression in response to salinity and osmotic stresses [7]. In rice, it has been reported that both *OsARF11* and *OsARF15* show differential expression in salt stress conditions, suggesting that they are involved in the rice response to stress [36]. Some of the tomato *SlARF* genes (*SlARF1*, *SlARF4*, *SlARF6A*, *SlARF7A*, *SlARF8A*, *SlARF9A*, *SlARF18*, *SlARF19*) can respond at the transcriptional level to abiotic stresses [47]. In addition, accumulating lines of evidence have indicated the involvement of *ARF* genes in abiotic stresses and related signal transduction pathways [1]. For example, *OsARF16* is required for the phosphate starvation response in rice, and knocking out *OsARF16* leads to primary roots, lateral roots and root hairs losing sensitivity to Pi deficiency [17]. Physic nut is considered to be extremely tolerant to salt and drought [19–20]. However, no information was previously available about the response of physic nut *ARF* genes to these stimuli. In our work, RNA-seq data generated from physic nut subjected to abiotic stresses, together with our qRT-PCR analysis, allowed us to identify *JcARF* genes involved in stress responses. The results suggested that seven of the 17 *JcARF* genes were induced or inhibited by salt and eight were induced or inhibited by drought (Figs 8 and 9). For example, *JcARF5*, *11*, *14* were down-regulated under salinity stress treatment compared with the control, whereas *JcARF2* and *12* were up-regulated after salinity stress, and *JcARF1* and *16* were up-regulated under drought stress treatment (Fig 8). Collectively, our findings suggest that these *JcARF* genes may play important roles in responses to abiotic stresses in physic nut, and they provide many candidates for subsequent study; their functions merit further investigation. In summary, although it is not yet possible to report the exact functions of the *JcARF* genes and their products, the analysis of expression profiles together with the conserved motifs presented in this paper form a valuable basis for future research in this area.

## Conclusion

In this study, a total of 17 *JcARF* genes were identified in the genome of physic nut, and the study provides the first comprehensive evaluation within the Euphorbiaceae of the phylogenetic relationships, exon-intron structure, conserved motif, chromosomal localization, potential targets for small RNAs, and expression profiles under normal growth, IAA and abiotic stress treatment conditions, of ARF family members. Phylogenetic and homolog comparison analysis of physic nut, rice and *Arabidopsis*, together with the expression profiles of *ARF* genes under various conditions, provide a useful foundation for future study aimed at understanding the potential role of each *JcARF* gene in regulating the growth and development of physic nut, and the responses of this species to auxin and abiotic stresses.

## Supporting information

S1 Table Primers used in this study. (XLSX)

S2 Table Prediction of MiR160, 167 and TAS3 target sites in *JcARF* genes. (DOC)

S3 Table Signal strength values for the expression of 17 *JcARF* genes in leaves under drought and salinity stresses based on RNA-seq. (XLSX)
