# Supplementary material for: Genome-wide identification and expression profiling of the auxin response factor (ARF) gene family in physic nut
Source: PLoS One. 2018 Aug 1;13(8):e0201024. doi: 10.1371/journal.pone.0201024 (PMC6070241; doi:10.1371/journal.pone.0201024)
Supplement: S2 Table — (DOC) [file pone.0201024.s002.DOC]

**Table S3. Predicted *miR160*, *167* and *TAS3* target sites in *JcARF* genes**

(A)

*JcARF1* 2515bp GCAGGCAUACAGGGAGCCAGGCAU 2538bp

*JcARF6* 1306bp GCUGGCAUGCAGGGAGCCAGGCAA 1328bp

*JcARF9* 1357bp GCUGGCAUGCAGGGAGCCAGGCAU 1380bp

*JcARF15* 1336bp GCAGGCAUACAGGGAGCCAGGCAU 1359bp

:|:||||||||||||||||||

*Jca-miR160a* 3’---ACCGUAUGUCCCUCGGUCCGU---5’

(B)

*JcARF3* 2430bp GAUAGAUCAGGCUGGCAGCUUGUAU 2452bp

*JcARF12* 2305bp CAUAGAUCAGGCUGGCAGCUUGUAU 2329bp

:||||||:|||||||||||

*Jca-miR167a* 3’---AUCUAGUACGACCGUCGAAGU---5’

(C)

*JcARF2* 1027bp ---GAGGAGUCUGGAAGACCCUCUAAGGUCUUGCAAGGUCAAGGUCAAGA..180bp..UUCCGAAGGUCUUGCAAGGUCAAGAAAUAUGCCCACUGAGAUCACUGACUGCAAAAGGUGAUUUCAAUCUGGGU----------- 1320bp

*JcARF4* 1285bp ---GGGGAAUCUUCAAGGUUCCAGAAGGUCUUGCAAGGUCAAGAAAUUUU..177bp..UCAACGAGGUCUUGCAAGGUCAAGAAAUAAUUCCAAGUUUGCCAUUUGGACGAGGCCUAAAAACUAAUGAGGCUUGUGA------ 1586bp

:|||:|||:|||||||||||| ||||:|||:||||||||||||

*Jca-TAS3*  3' CUCCGGAAUGUUCCAGUUCUU 5' 3' CUCCGGAAUGUUCCAGUUCUU 5'

|||||||||||||||||||||

*pre-tasiR-ARF*(RC) 723bp **-----GGAAUGCUCAGAAGCAGUGAGAGCCUUACAAGGUCAAGAA-------------------GAGGCCUUACAAGGUCAAGAA---GCAAUUAACUCGUAUGAGAUCCCAGUUAACAGAGUUACGAGUCAAGCUCUCCGCCA** 594bp

(A) Sequences of miR160 target sites in *JcARF* mRNAs. Base pairings to the *Jca-miR160a* sequence are shown.

(B) Sequences of miR167 target sites in *JcARF* mRNAs. Base pairings to the *Jca-miR167a* sequence are shown.

(C) Alignment of maize tasiR-ARF sequences and the potential *JcARF* target genes. To visualize similarity, the sense orientation of each target gene sequence was aligned with the reverse complement (RC) of the physic nut *pre-tasiR-ARF* sequences. The (-) character marks the gap opened to allow the closest possible alignment between the multiple *JcARF* sequences. Nucleotides of physic nut *pre-tasiR-ARF* are shown in boldface. Regions of sequence similarity between the 21-nt *pre-tasiR-ARF* (shaded with gray) and the ARF target genes are boxed. The potential target gene mRNAs aligned closely with *Jca-TAS3* (red and blue letters), with two to four mismatches. The target gene name and the location of the recognition sequence are indicated (note that all of physic nut genes, with two *Jca-TAS3* recognition sites).
